# Epstein-Barr Virus-Driven B-Cell Transformation under Germinal Center Hypoxia Requires External Unsaturated Fatty Acids

**DOI:** 10.21203/rs.3.rs-6506954/v1

**Published:** 2025-04-24

**Authors:** Larissa Havey, Haixi You, John M. Asara, Yin Wang, Rui Guo

**Affiliations:** Department of Molecular Biology and Microbiology, Tufts University, Boston, MA 02111; Department of Molecular Biology and Microbiology, Tufts University, Boston, MA 02111; Division of Signal Transduction, Beth Israel Deaconess Medical Center and Department of Medicine, Harvard Medical School, Boston, MA 02115; Division of Infectious Diseases, Department of Medicine, Brigham and Women’s Hospital, 181 Longwood Avenue, Boston, MA 02115; Department of Molecular Biology and Microbiology, Tufts University, Boston, MA 02111

## Abstract

Epstein-Barr virus (EBV) contributes to over 200,000 cancers annually, predominantly aggressive lymphomas originating from hypoxic germinal centers (< 1% O_2_). However, conventional models fail to recapitulate the physiologically relevant hypoxic microenvironment which profoundly influences B-cell metabolic remodeling during transformation. Here, we establish an *ex vivo* model of EBV-driven B-cell transformation under 1% O_2_, demonstrating robust transformation and super-enhancer activation of oncogenic regulators, including MYC. Multi-omic analyses reveal distinct metabolic adaptations to hypoxia. Unlike normoxic B-cells, which rely on fatty acid desaturases and oxidation to mitigate lipotoxicity, hypoxically transformed B-cells suppress fatty acid synthesis while upregulating glycerophospholipid metabolism and lipid droplet formation to buffer excess saturated lipids. Consequently, these cells exhibit heightened dependence on external unsaturated fatty acids to support proliferation. Our findings provide the first physiologically relevant *ex vivo* model of EBV-driven B-cell transformation under hypoxia, uncovering metabolic vulnerabilities that could inform targeted therapeutic strategies for EBV-associated malignancies.

## Introduction

Epstein-Barr Virus (EBV) is a gammaherpesvirus that can transform human naïve B-cells into lymphoblastoid cell lines (LCLs) in culture. It infects approximately 90% of adults globally^1^, and is implicated in various malignancies, including B-cell lymphomas like Diffuse Large B-cell Lymphomas (DLBCL), Burkitt Lymphomas (BL), and classic Hodgkin Lymphomas (cHL), as well as epithelial cancers such as nasopharyngeal and gastric carcinomas^2–7^. Due to its transformative capability, immunocompromised individuals, particularly those with HIV or undergoing post-transplant immunosuppressive treatments, are at an increased risk for developing these EBV-associated lymphoproliferative diseases and lymphomas^8, 9^.

EBV encodes Epstein-Barr nuclear antigens (EBNAs) and latent membrane protein (LMPs) to transform B-cells. During the initial pre-latency phase, EBV expresses the Epstein-Barr virus nucleus antigen 2 (EBNA2) and EBNA-leader protein (EBNA-LP)^10–14^. EBNA2 transactivates c-MYC, a proto-oncogene by assembling a super-enhancer upstream of c-MYC gene loci^15, 16^. EBNA2 and c-MYC transcriptionally remodel B-cell metabolism including aerobic glycolysis, oxidative phosphorylation (OXPHOS), mitochondrial one-carbon metabolism, mevalonate and fatty acid biosynthesis prior to the first mitosis, which metabolically prepares B-cells for upcoming hyperproliferation stages^14, 17, 18^. Starting from day 4 post-infection (DPI) the virus processes into the Latency IIb program. This stage is marked by the expression of all six EBNA proteins (EBNA1, 2, LP, 3A, 3B, and 3C) and BL-like hyperproliferation. Around 7 DPI, EBNA2 activates the bidirectional promotor of the latent membrane protein 1 and 2 (LMP1 and LMP2) that take over the transformation processes that simulate key growth pathways in B-cells^19^. LMP1 functions as a constitutive CD40 receptor analog, activating NF-κB, JAK/STAT, and PI3K/AKT pathways to promote cell survival and proliferation. LMP2A mimics activated B-cell receptor signaling, maintaining latency and supporting cell growth by modulating PI3K/AKT and MAPK pathways^20, 21^.

**Figure 1:**
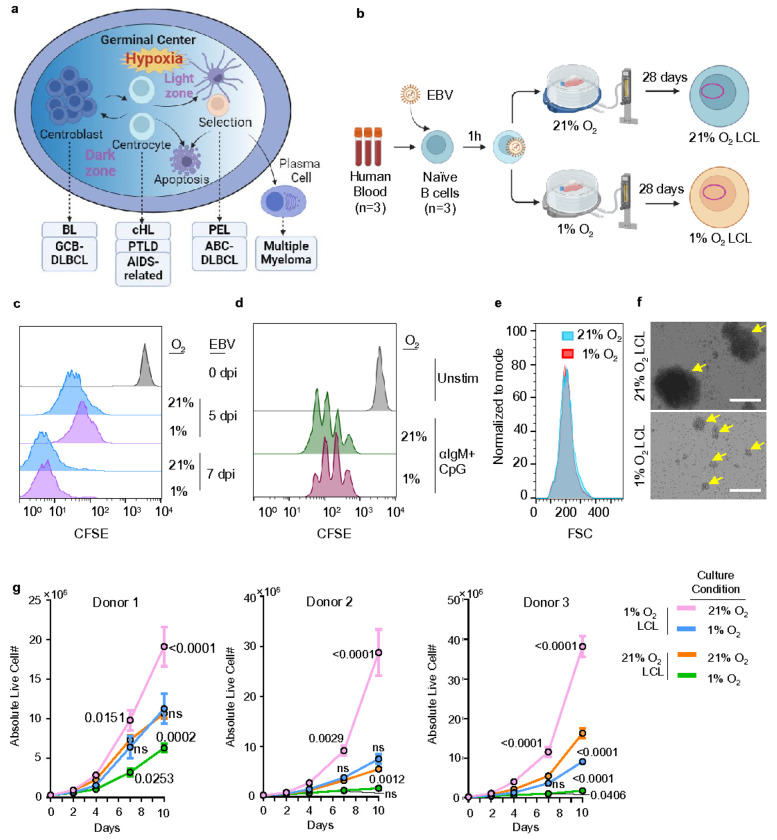
EBV transforms human primary B-cells under 1% O_2_. a. A schematic picture showing the GC origin of EBV-associated lymphomas. GC represents a physiologically relevant hypoxic microenvironment. BL, Burkitt Lymphoma; GCB-DLBCL, Germinal Center B-cell-like Diffuse Large B-cell Lymphoma; cHL,Classical Hodgkin Lymphoma; PTLD, Post-Transplant Lymphoproliferative Disorder; AIDS-related – AIDS-related Lymphoma; PEL, Primary Effusion Lymphoma; ABC-DLBCL, Activated B-cell-like Diffuse Large B-cell Lymphoma b. A schematic picture of the new *ex vivo* model of EBV B-cell transformation under hypoxia. c. CFSE staining analysis of human primary B-cells transformed by EBV at a MOI of 0.1 under 1% or 21% O_2_ for 5 or 7 days. This is a representative FACS histogram plot from n=3 experiments. d. CFSE staining analysis of human primary B-cells stimulated by a combination of 1μg/mL anti-human IgM IgG and 0.5 μM CpG under 1% or 21% O_2_ for 5 days. This is a representative FACS histogram plot from n=3 experiments. e. Forward scatter (FSC) analysis of 1% or 21% O_2_ LCLs. This is a representative FACS histogram plot from n=3 experiments. f. Representative microscopic picture of 1% or 21% O_2_ LCLs. Arrows indicate the clumps in the LCL culture. Scale bar, 200 μM. g. Growth curve of 1% O_2_ LCLs and 21% O_2_ LCLs. Each LCL was split into two flasks, with one incubated at 1% O_2_ and the other at 21% O_2_. Mean +/− SEM values are from n=3 experiments. P values were determined by using two-way ANOVA with Dunnett’s test. LCLs derived from different donors are plotted separately.

The GC model of persistent EBV infection suggests that EBV-infected cells mirror the developmental pathway of uninfected B lymphocytes as they differentiate into memory B-cells^22^.The GC is composed of a dark zone, where B-cells called centroblasts proliferate and undergo somatic hypermutation, and a light zone, composed of non-dividing B-cells called centrocytes^23^. After GC entry, EBV-infected B-cells may transition from Latency III to Latency II or Latency I. This transition reflects a gradual reduction in viral gene expression, helping the infected cells evade immune detection. Specifically, EBV-infected B-cells transitioning to the memory B-cell phenotype often establish Latency I, characterized by the exclusive expression of EBNA1^7^. Interestingly, most EBV-associated B-cell lymphomas are of GC B-cell origin^24^(Fig. 1a), indicating that the lymphoid GC microenvironment might play a previously unrecognized role in EBV-driven tumorigenesis.

Oxygen tension naturally decreases across the human body, starting from arterial blood (~ 13%), moving through vital organs (~ 6%; including the brain, liver, and lungs), and reaching hypoxia in bone marrow and secondary lymphoid tissues (< 3%; notably in lymph nodes)^25, 26^. Remarkably, GCs in secondary lymphoid organs show oxygen tensions beneath 1%, as has been precisely determined using hypoxyprobe labeling, which enables the sensitive visualization of hypoxia in situ when oxygen levels fall below 1%^27, 28^. Studies found that hypoxia strongly impacts B-cell activation in the GC light zone, significantly influencing B-cell development^29^. *In vivo* CRISPR screens highlighted that the GC hypoxic microenvironment can shape the function of T follicular helper cells and B-cell fate decisions^30^.

Similarly, because EBV co-opts many of the same signaling networks used by uninfected B-cells in the GC^7, 31^, the profound hypoxia that shapes normal B-cell activation may also be integral to viral-driven transformation and tumorigenesis. Traditional *ex vivo* transformation models have provided valuable insights into EBV biology,^24, 31, 32^ but fail to replicate critical aspects of the hypoxic GC microenvironment. Particularly, these models may overlook the extensive metabolic reprogramming required for B-cell transformation under hypoxic conditions. Nevertheless, the metabolic pathways that are rewired under hypoxia may represent key vulnerabilities in EBV transformation or EBV-associated cancers. Investigating these hypoxia-driven metabolic pathways could provide new therapeutic targets for disrupting EBV-mediated oncogenesis.

To model EBV-driven B-cell transformation under physiologically relevant hypoxia, we developed a novel *ex vivo* transformation system under GC hypoxia. Integrated transcriptomic, metabolomic, and lipidomic analyses highlighted that hypoxically transformed LCLs suppress fatty acid synthesis, relying on extracellular unsaturated fatty acids for proliferation. Upregulated lipid droplet formation sequesters excess saturated lipids, mitigating lipotoxicity and supporting survival under physiologically relevant hypoxia, highlighting altered lipid supply as a potential vulnerability in EBV-associated malignancies.

## Results

### EBV transforms human primary B-cells under 1% O_2_ tension.

In our new *ex vivo* transformation model (Fig. 1b), freshly isolated CD19 + human resting B-cells are initially infected with the EBV strain B95.8 for one hour to standardize the initial viral entry across samples. Subsequently, these infected cells are divided equally into two groups: one group is cultured in a standard incubator with 21% O_2_, and the other under the hypoxic condition in an incubator with 1% O_2_. This setup mimics the natural infection process in immunocompromised patients who experience EBV lytic reactivation and viremia, where EBV-infected B-cells from peripheral blood migrate to the hypoxic environments of lymphoid organs. The cells are cultured for 28 days, during which time the EBV drives their transformation into LCLs^14, 33, 34^. A similar hypoxic condition has been used in Kaposi’s sarcoma-associated herpesvirus (KSHV) infection in SLK cells^35^. We observed that under 1% O_2_ conditions, EBV can comparatively transform human primary B-cells; CellTrace CFSE assay showed that EBV-driven B-cell proliferation under 1% O_2_ is comparable to that under 21% O_2_ during the first 7 days (Fig. 1c). Similarly, a combination of mitogens, which use anti-human IgM IgG to stimulate B-cell receptor (BCR) pathway and CpG to stimulate Toll-like receptor 9 (TLR9) pathway can activate B-cell proliferation under GC hypoxia (Fig. 1d). This suggests that both EBV-driven and mitogen-induced B-cell activations are not hindered by low oxygen levels.

By 28 DPI, LCLs transformed under 1% O_2_ (hereafter referred to as 1% O_2_ LCLs) exhibited comparable cell sizes to their normoxic counterparts, as assessed by forward scatter (FSC) in flow cytometry (Fig. 1e). Notably, 1% O_2_ LCLs formed clumps in culture, but to a much lesser extent in terms of size compared to the clumping observed in classical 21% O_2_ LCLs^36, 37^(Fig. 1f). 1% O_2_ LCLs demonstrated significantly higher proliferation rates than 21% O_2_ LCLs when both were cultured under 1% O_2_ conditions (Fig. 1g), indicating superior adaptation to hypoxia that facilitates more robust growth. Strikingly, upon shifting to a 21% O_2_ environment, 1% O_2_ LCLs exhibited even greater proliferation rates than 21% O_2_ LCLs under the same conditions (Fig. 1g). Since 1% O_2_ LCLs and 21% O_2_ LCLs share the same genetic background, these findings suggest that EBV establishes a specialized transformation program tailored to the hypoxic GC microenvironment for enhanced proliferation and survival. Of note, this growth advantage was consistently observed across 1% O_2_ LCLs transformed from different donors (Fig. 1g).

### Hypoxia has little impact on EBV oncogene expression and super-enhancer establishment

EBV employs a precisely controlled program to regulate B-cell transformation^10^. Upon infection, the EBV genome rapidly becomes chromatinized, expressing viral oncogenes and activating host regulatory networks critical for cell survival and proliferation^38^. To examine how oxygen availability influences key regulators of this transformative process, we assessed EBV oncogene expression in newly infected B-cells under 21% O_2_ and 1% O_2_ conditions.

**Figure 2:**
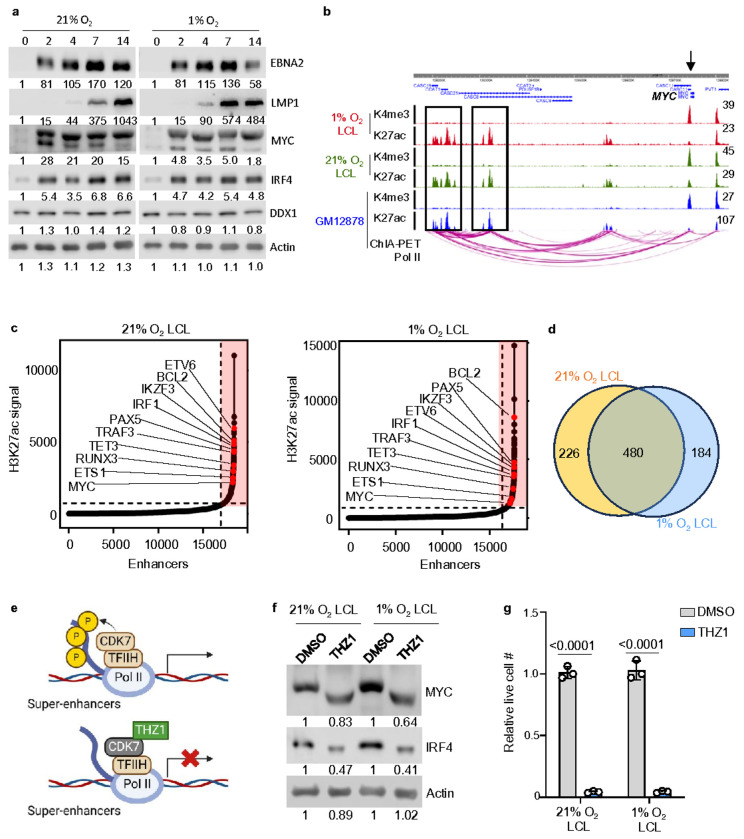
Hypoxia has minimal effect on EBV oncogene expression and the establishment of super-enhancers. a. Immunoblot analysis for indicated proteins in whole cell lysates (WCL) in EBV newly transformed human primary B-cells, collected at indicated days post-infection in either 1% or 21% O_2_. This is a representative experiment from donor 3. Analyses from another donor is in Extended Data Figure S1. b. H3K27ac and H3K4me3 ChIP-seq tracks from 1% O_2_, 21% O_2_, or GM12878 LCL, and GM12878 ChIA-PET Pol ll tracks are shown. The black arrow indicates MYC loci. Black boxes indicate SEs. c. Enhancers are ranked by their H3K27ac ChIP-seq signals in either 1% or 21% O_2_ LCLs. The inflection point on the plotted curve was then selected as the cutoff to separate SEs from typical enhancers. SE associated genes are indicated in red box, selected of which are linked to their direct target genes by H3K27ac HiChIP. d. Venn diagram analysis of SE-associated genes in 1% or 21% O_2_ LCLs. e. A schematic picture showing THZ1 inhibits CDK7 at SEs to suppress SE target gene expression. f. Immunoblot analysis of MYC, IRF4, and Actin from 1% or 21% O_2_ LCLs treated with DMSO or 100nM THZ1 for 48 h. Representative blot of n = 3 replicates shown. g. Relative live cell number in 1% or 21% O_2_ LCL treated with DMSO or 100 nM THZ1 for 4 days. Mean +/− SD values were from n=3 experiments. P-values were calculated by two-way ANOVA with Sidak’s multiple comparisons test.

EBNA2, an essential transcription factor for EBV-driven B-cell outgrowth^39^, exhibited a similar expression pattern at 2 DPI across both oxygen conditions, suggesting that its activation is largely oxygen-independent (Fig. 2a, **Extended Data** Fig. 1a). A major function of EBNA2 is the transactivation of MYC, a key proto-oncogene that regulates cell metabolism^40–43^. Together, EBNA2 and MYC orchestrate B-cell metabolic reprogramming to fuel transformation^17, 44^. Consistent with EBNA2’s oxygen-independent expression, we observed that MYC expression peaked at 2 DPI in both 1% and 21% O_2_ conditions; however, its relative expression level at 2 DPI was notably lower under hypoxia (Fig. 2a, **Extended Data** Fig. 1a). Interestingly, we observed an accelerated induction of LMP1 under 1% O_2_, occurring earlier than its 21% O_2_ counterpart), a trend that was consistently observed across different donors (Fig. 2a, **Extended Data** Fig. 1a). The earlier induction of LMP1 under hypoxia may be linked to the observed reduction in MYC expression (Fig. 2a, **Extended Data** Fig. 1a), as previous findings indicate that c-MYC represses LMP1 transcription in EBV-infected lymphoma cells and LCL models^12^.

IRF4 is a key transcription factor in B-cell differentiation^45^ and it plays a key role in maintaining EBV latency and B-cell survival^46, 47^. In EBV-infected B-cells, IRF4 and BATF jointly repress tumor suppressors like PRDM1 and BCL2L11, thereby promoting transformation^47^. Given its role in EBV transformation, we assessed its expression under 1% and 21% O_2_ conditions. Our analysis revealed that IRF4 was induced under both 1% and 21% O_2_ conditions without notable differences (Fig. 2a, **Extended Data** Fig. 1a).

Super-enhancers (SEs) are clusters of highly active enhancers that regulate key genes driving cell identity and malignancy^48, 49^. Compared with typical enhancers, SEs have larger size, higher transcription factor occupancy, and greater sensitivity to perturbation^48, 49^. In LCLs, chromatin immunoprecipitation followed by sequencing (ChIP-seq), identified that EBNA2, LMP1, and NF-κB-driven SEs co-activate the expression of proto-oncogenes such as MYC and IRF4^50^. Interestingly, hypoxia is known to influence SE activity through hypoxia-inducible factor (HIF)-mediated transcriptional regulation^51^, but its impact on EBV-driven SEs remains unexplored. To address this, we performed H3K27ac ChIP-seq (to profile SE activity) and H3K4me3 ChIP-seq (to analyze promoter activity) in 1% or 21% O_2_ LCLs. Our heatmap analysis revealed highly similar H3K4me3 and H3K27ac enrichment patterns between 1% and 21% O_2_ LCLs across transcription start sites (TSS) and transcription end sites (TES) (**Extended Data** Fig. 1b). We further examined key EBV-driven SE targets, including MYC, IRF4, and BCL2, and found no notable differences in their peak shapes and intensities between 1% and 21% LCLs, also with profiles comparable to those observed in GM12878 LCLs (Fig. 2b and **Extended Data** Fig. 1c). To assess the EBV-driven SEs in 1% and 21% O_2_ LCLs, we performed Homer SE analysis using H3K27ac ChIP-seq data. SE-associated genes were ranked based on H3K27ac signal intensity, revealing a highly similar SE landscape in 1% and 21% O_2_ LCLs (Fig. 2c **and SE analyses are in Source Data** Fig. 2c). Key factors driving LCL survival and transformation, including MYC proto-oncogene (*MYC*), ETS proto-oncogene 1 (*ETS1*), RUNX family transcription factor 3 (*RUNX3*), tet methylcytosine dioxygenase 3 (*TET3*), TNF receptor-associated factor 3 (*TRAF3*), interferon regulatory factor 1 (*IRF1*), IKAROS family zinc finger 3 (*IKZF3*), paired box 5 (*PAX5*), and BCL2 apoptosis regulator (*BCL2*), were consistently associated with SEs under both conditions (Fig. 2c). Notably, the Venn diagram analysis showed substantial overlap in SE-associated genes between 1% and 21% O_2_ LCLs, with 480 shared SE-associated genes (Fig. 2d). These results indicate that EBV establishes a robust SE landscape that remains largely stable under hypoxia, supporting LCL growth and survival across oxygen conditions.

Given these observations, we next investigated whether EBV-driven SEs remain viable therapeutic targets under hypoxic conditions^15^. SEs drive high-level transcription of key oncogenes by recruiting transcriptional machinery, including cyclin-dependent kinase 7 (CDK7), a critical component of the transcription initiation complex TFIIH^52^. As CDK7 phosphorylates RNA polymerase II, it facilitates transcriptional pause release and elongation, making SE-driven genes particularly dependent on its activity^53^. THZ1, a selective CDK7 inhibitor, disrupts this process by blocking transcriptional activation at SE-regulated loci, leading to preferential suppression of oncogenes with SE dependency^52^ (Fig. 2e). Accordingly, treatment with THZ1 effectively downregulated MYC and IRF4 expression (Fig. 2f) and induced a significant growth defect in LCLs cultured under both 1% and 21% O_2_ conditions (Fig. 2g). Collectively, our data indicates that EBV-driven oncogene expression and SEs are resilient to hypoxic conditions, ensuring sustained LCL growth and survival across varying oxygen levels.

### RNA-seq Reveals Hypoxia-Specific Transcriptomic Adaptations in 1% O_2_ LCLs

**Figure 3:**
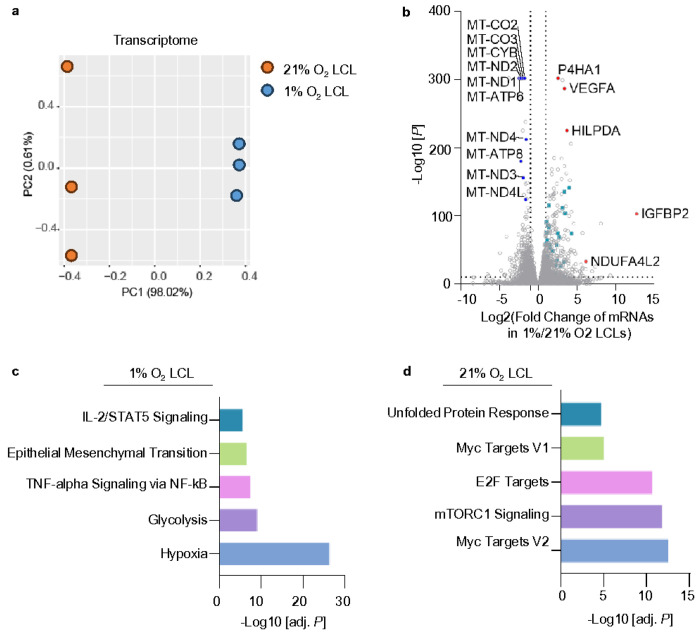
RNA-seq Reveals Hypoxia-Specific Transcriptomic Adaptations in 1% O_2_ LCLs a. PCA analysis of transcriptome identified by RNAseq in 1% O_2_ LCLs (blue) or 21% O_2_ LCLs (orange), across n=3 replicates. b. Volcano plot visualization of -log_10_ (p-value statistical significance) vs log_2_ (mRNA abundance foldchange) from triplicate RNAseq analysis of 1% vs 21% O_2_ LCLs. Cyan, genes related to glycolysis. Common hypoxia-responsive genes are highlighted in red. c. Enrichr pathway analysis of gene sets significantly upregulated in 1% O_2_ LCLs. Shown are the -log_10_ (adjusted p-values) from Enrich analysis of triplicate RNAseq datasets. d. Enrichr pathway analysis of gene sets significantly upregulated in 21% O_2_ LCLs. Shown are the -log_10_ (adjusted p-values) from Enrich analysis of triplicate RNAseq datasets.

To explore the transcriptional changes associated with hypoxia during EBV transformation, RNA sequencing (RNA-seq) was performed on 1% O_2_ and 21% O_2_ LCLs. Principal component analysis (PCA) of the transcriptome demonstrated distinct clustering of samples based on oxygen tension, with PC1 accounting for 98.02% of the variance (Fig. 3a). This clear segregation highlights the extensive reprogramming of transcriptional profiles in response to hypoxia. Differentially expressed gene (DEGs) analysis revealed a significant number of hypoxia-induced genes with p-values < 0.05 and fold change > 2 (Fig. 3b, **RNAseq data is in Source Data** Fig. 3b). Notably, hallmark hypoxia-responsive genes such as prolyl 4-hydroxylase subunit alpha 1 (*P4HA1*), vascular endothelial growth factor A (*VEGFA*), and hypoxia-inducible lipid droplet-associated protein (*HILPDA*) showed marked upregulation under 1% O_2_ conditions. Additional genes, including NDUFA4 mitochondrial complex associated like 2 (*NDUFA4L2*), which downregulates mitochondrial OXPHOS activity to reduce oxidative stress, optimizing survival in low-oxygen environments^54^, were also upregulated (Fig. 3b). Conversely, mitochondrial DNA encoded genes, including *MT-ND* (Complex I), *MT-CYB* (Complex III), *MT-CO* (Complex IV), and *MT-ATP* (ATP synthase), were significantly downregulated under hypoxia, suggesting a suppression of OXPHOS (Fig. 3b). Of note, glycolytic genes (highlighted in cyan in Fig. 3b) were prominently upregulated in 1% O_2_ LCLs.

Gene set enrichment analysis revealed pathways specific to the hypoxic condition in 1% O_2_ LCL. Terms such as glycolysis, epithelial-mesenchymal transition, and IL-2/STAT5 signaling were significantly enriched from upregulated DEGs under 1% O_2_. The activation of glycolysis pathways aligns with the known metabolic switch to anaerobic energy production under hypoxia^55^ (Fig. 3c). In contrast, 21% O_2_ LCLs exhibited enrichment in pathways related to the unfolded protein response, MYC signaling, and mTORC1 activity, highlighting metabolic and signaling processes that are more prominent under oxygen-rich environments (Fig. 3d).

To gain further insights into how 1% vs 21% O_2_ impacts EBV-driven B-cell metabolism, we next analyzed metabolic gene responses. DEGs upregulated in 1% O_2_ LCLs were filtered within this metabolic gene set^56^. Subsequently, STRING, a protein-protein interaction networks functional enrichment analysis, was conducted using metabolic DEGs to depict the metabolic network specific to the 1% O_2_ LCLs (**Extended Data** Fig. 2). Our analysis highlights a central role of glycolysis, with key genes such as aldolase, fructose-bisphosphate A (*ALDOA*), hexokinase 2 (*HK2*), 6-phosphofructo-2-kinase/fructose-2,6-biphosphatase 3 (*PFKFB3*), and lactate dehydrogenase A (*LDHA*) forming highly connected hubs, reflecting a metabolic shift toward anaerobic glycolysis as the primary pathway for ATP production. Notably, *HK2* and *PFKFB3* act as critical regulatory nodes linking upstream hypoxia-induced signaling to downstream glycolytic processes^57, 58^.

The network demonstrates extensive cross-talk between glycolysis and lipid metabolism, with upregulated genes, Insulin induced gene 2 (*INSIG2*) and sterol regulatory element binding transcription factor 1 (*SREBF1*), coordinating lipid biosynthesis and storage via lipid droplet formation^59^ (**Extended Data** Fig. 2). SREBF1 is a key regulator of lipid metabolism^59, 60^. Its activation ensures adequate lipid production for energy storage and membrane formation^59^. INSIG2 acts as a crucial regulator of SREBF1 activation, controlling its release from the endoplasmic reticulum (ER) in response to lipid levels^61^. When cellular lipid stores are sufficient, INSIG2 prevents SREBF1 activation, thereby reducing lipid synthesis^61^.

Additionally, genes involved in branched-chain amino acid (BCAA) catabolism, such as branched chain aminotransferase 1 (*BCAT1*), connect glycolysis with anaplerosis, maintaining TCA cycle activity under oxygen limitation (**Extended Data** Fig. 2). These interactions highlight the metabolic flexibility of 1% O_2_ LCLs, which may utilize both carbohydrates and amino acids to sustain growth and adapt to fluctuating environmental conditions. Together, these metabolic adaptations underscore the ability of EBV-transformed LCLs to thrive in hypoxic environments, exploiting these niches to sustain growth and survival.

### Metabolic Adaptations Highlight Glycolysis and Redox Shifts in 1% O_2_ LCLs

**Figure 4:**
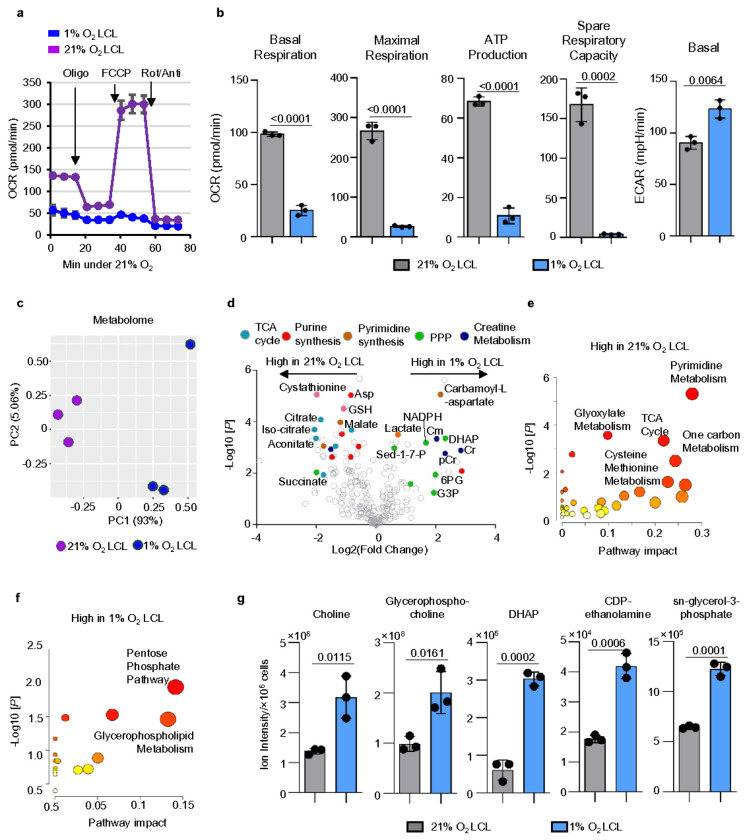
Hypoxia-Driven Metabolic Shifts Reshape Energy and Redox Balance in EBV-Transformed LCLs a. Mitochondrial stress test of 1% or 21% O_2_ LCLs using a Seahorse analyzer. Both cells were seeded at 3×10^5^/mL. Mean +/− SD values were from n=3 experiments. b. Seahorse OCR linked to basal respiration, maximal respiration, and ATP production and basal ECAR in 1% or 21% O_2_ LCLs. Mean +/− SD values were from n=3 experiments. P-values were calculated using an unpaired Student’s t-test. c. PCA of metabolome identified by LC/MS metabolomic analysis in 1% O_2_ LCLs (blue) or 21% O_2_ LCLs (purple), across n=3 replicates. d. Volcano plot visualization of -Log_10_ (p-value statistical significance) vs Log_2_ (metabolite abundance foldchange) from triplicate metabolomic analysis of 1% vs 21% O_2_ LCLs. e. Metabolic pathway analysis highlighting pathways significantly upregulated in 21% O_2_ LCLs. The x-axis shows pathway impact values from MetaboAnalyst 3.0 topological analysis; the y-axis shows -log_10_ of P-value from pathway enrichment analysis. f. Metabolic pathway analysis highlighting pathways significantly upregulated in 1% O_2_ LCLs. The x-axis shows pathway impact values from MetaboAnalyst 3.0 topological analysis; the y-axis shows -log_10_ of P-value from pathway enrichment analysis. g. Bar chart analysis of ion intensity of intermediates of glycerophospholipid metabolism in 1% or 21% O_2_ LCLs. Mean +/− SD values were from n=3 experiments. P-values were calculated using an unpaired Student’s t-test.

To assess mitochondrial activity, we performed a Seahorse analysis to measure the oxygen consumption rate (OCR) in 1% and 21% O_2_ LCLs. 1% O_2_ LCLs exhibited significantly lower basal and maximal respiration, ATP production, and spare respiratory capacity (Fig. 4a–b). Notably, this suppression of mitochondrial respiration is not due to reduced mitochondrial biogenesis; paradoxically, MitoTracker Green staining revealed significantly higher mitochondrial content in 1% O_2_ LCLs (**Extended Data** Fig. 3a), suggesting a potential compensatory response, as previously reported^62–64^. Consistent with RNA-seq data, the extracellular acidification rate (ECAR) was significantly elevated in 1% O_2_ LCLs, indicating a shift toward glycolysis (Fig. 4b).

We next performed liquid chromatography-mass spectrometry (LC/MS) analysis to compare metabolome in 1% and 21% O_2_ LCLs. PCA analysis revealed distinct metabolic shifts, with PC1 accounting for 93% of the variance, demonstrating clear clustering based on oxygen conditions (Fig. 4c). 21% O_2_ LCLs displayed higher levels of TCA cycle intermediates, reflecting their greater reliance on mitochondrial oxidative metabolism (Fig. 4d–e **and metabolomics data is in Source data** Fig. 4c-d). Notably, key metabolites in one-carbon metabolism and pyrimidine synthesis were enriched, suggesting enhanced nucleotide biosynthesis to support rapid proliferation. These metabolic features are consistent with the other conventional EBV transformation models, where oxygen availability enables mitochondrial ATP generation and biosynthetic pathways necessary for biomass accumulation^14, 17^.

In contrast, 1% O_2_ LCLs exhibited increased levels of glycolytic and pentose phosphate pathway (PPP) intermediates (Fig. 4d). We observed a significantly elevated NADPH/NADP^+^ ratio in 1% O_2_ LCLs, suggesting a shift in redox balance to counteract hypoxia-induced reactive oxygen species (ROS) (**Extended Data** Fig. 3b). While heightened PPP activity is one possible contributor^65, 66^, treatment with 6-aminonicotinamide (6-AN), a glucose-6-phosphate dehydrogenase (G6PD) inhibitor, had little selective inhibition on 1% O_2_ LCL growth (**Extended Data** Fig. 3c). This suggests that alternative NADPH-generating pathways, such as the malic enzyme (ME1), and isocitrate dehydrogenase 1 (IDH1) may compensate for PPP activity in maintaining redox homeostasis^67, 68^.

Notably, the NADH/NAD^+^ ratio remained unchanged. Given the increased lactate production and the elevation of *LDHA*, this homeostasis may be maintained through efficient NAD^+^ regeneration via fermentation. Additionally, creatine metabolism was significantly upregulated in 1% O_2_ LCLs, consistent with its previously reported role as a rapid ATP buffer system during hypoxia^69, 70^ (Fig. 4d).

Of interest, glycerophospholipid metabolism was elevated in 1% O_2_ LCLs (Fig. 4f). 1% O_2_ LCLs exhibited markedly increased levels of choline, glycerophosphocholine, dihydroxyacetone phosphate (DHAP), CDP-ethanolamine, and sn-glycerol-3-phosphate compared to 21% O_2_ LCLs (Fig. 4g). These metabolites are essential intermediates in the biosynthesis of phosphatidylcholine (PC) and phosphatidylethanolamine (PE), major components of cellular membranes. Notably, the elevation of DHAP and sn-glycerol-3-phosphate—key intermediates linking glycolysis to triglyceride (TG) synthesis—suggests a broader metabolic shift beyond membrane remodeling.

### Hypoxia induces triglyceride storage and lipid droplet formation in 1% O_2_ LCLs.

**Figure 5:**
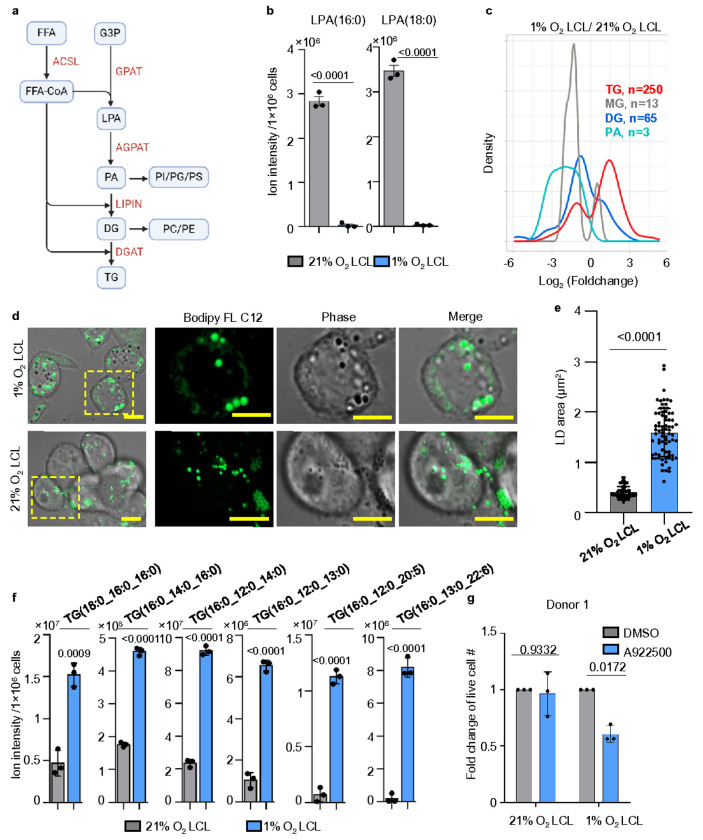
Hypoxia induces TG biosynthesis and lipid droplet formation in 1% O_2_ LCLs. a. A schematic picture showing phospholipids and TG biosynthetic pathway. FFA, free fatty acids; FFA-CoA, free fatty acid coenzyme A; G3P, sn-glycerol-3-phosphate; LPA, lysophosphatidic acid; PA, phosphatidic acid; PI, phosphatidylinositol; PG, phosphatidylglycerol; PS, phosphatidylserine; DG, diacylglyceride; PC, phosphatidylcholine; PE, phosphatidylethanolamine. b. Bar chart analysis of ion intensity of indicated LPA species in 1% or 21% O_2_ LCLs. Mean +/− SD values were from n=3 experiments. P-values were calculated using an unpaired Student’s t-test. c. Density plot analysis of log_2_ (lipid abundance foldchange) of indicated lipid species from triplicate lipidomic analysis of 1% vs 21% O_2_ LCLs. d. Confocal microscopic analysis of 1% vs 21% O_2_ LCLs treated with 10 μM Bodipy FL C12 for 20 min. Representative of n=3 experiments. Scale bar, 5 μm. e. LD area analysis was performed on 1% and 21% O_2_ LCLs treated with 10 μM Bodipy FL C12 for 20 minutes. LD area was quantified using ImageJ from three randomly selected images, each containing approximately 5 cells. f. Bar chart analysis of ion intensity of indicated TG species in 1% or 21% O_2_ LCLs. Mean +/− SD values were from n=3 experiments. P-values were calculated using an unpaired Student’s t-test. g. Fold change of live cell number of 1% or 21% O_2_ LCL treated with DMSO or 10 μM of A922500, a DGAT1 inhibitor for 72 hours. Mean +/− SD values were from n=3 experiments. P-values were determined using two-way ANOVA with Sidak’s multiple comparisons test.

We next investigated whether 1% O_2_ LCLs rewire TG metabolism as an adaptive strategy to hypoxia. TG biosynthesis is a multi-step process, which plays a key role in hypoxia adaptation^71^. In this pathway, fatty acyl-CoA is first processed by glycerol-3-phosphate acyltransferase (GPAT) to form lysophosphatidic acid (LPA), which is further acylated by 1-acylglycerol-3-phosphate O-acyltransferase (AGPAT) to produce phosphatidic acid (PA). PA is then dephosphorylated by phosphatidate phosphatase (LIPIN) to generate diacylglycerol (DG), which is finally converted into TG by diacylglycerol O-acyltransferase (DGAT) (Fig. 5a).

Our lipidomic analysis revealed a significant depletion of lysophosphatidic acid (LPA) species, specifically LPA(16:0) and LPA(18:0), in 1% O_2_ LCLs (Fig. 5b), accompanied by a global accumulation of triglycerides (TG) (Fig. 5c **and lipidomic data is in Source Data** Fig. 5c). This shift suggests a metabolic adaptation favoring TG synthesis under hypoxia. This reprogramming likely serves two primary functions: (1) redirecting saturated fatty acids away from membrane lipid synthesis to mitigate lipotoxic stress, particularly as stearyl desaturase 1 (SCD1) and fatty acid oxidation (FAO) are inhibited due to limited O_2_ availability, and (2) increasing lipid storage capacity to buffer against energy fluctuations^71–73^. Notably, a recent preprint demonstrated the critical roles of SCD1 and fatty acid desaturase 2 (FADS2) in EBV-induced B-cell proliferation^74^.

TGs are stored in lipid droplets (LDs). RNA-seq analysis revealed significant upregulation of perilipin 2 (*PLIN2*) and perilipin 3 (*PLIN3*) in 1% O_2_ LCLs, while other *PLIN*s remained barely expressed (**Extended Data** Fig. 4a). PLIN2 stabilizes LDs and promotes TG accumulation, while PLIN3 facilitates lipid storage and trafficking^75^. Additionally, the upregulation of very low-density lipoprotein receptor (*VLDLR*) suggests increased lipid uptake (**Extended Data** Fig. 4a). To visualize LDs, we treated 1% O_2_ and 21% O_2_ LCLs cells with Bodipy FL C12, a saturated lipid probe and performed live-cell confocal microscopy. Interestingly, LDs in 1% O_2_ LCLs were significantly larger than those in 21% O_2_ LCLs (Fig. 5d and e), highlighting an increased capacity to convert lipids into TGs and store them within LDs.

Notably, TG species such as TG(16:0_16:0_16:0) and TG(18:0_16:0_16:0), composed entirely of saturated acyl chains, were significantly elevated in 1% O_2_ LCLs. Additionally, TGs incorporating both saturated and unsaturated fatty acids, including TG(16:0_12:0_20:5) and TG(16:0_13:0_22:6), were also significantly enriched (Fig. 5f). This supports the hypothesis that 1% O_2_ LCLs store saturated lipids in LDs as a protective strategy against lipotoxicity.

To further test this hypothesis, we treated 1% and 21% O_2_ LCLs with A922500, a selective DGAT1 inhibitor, to block TG biosynthesis. A922500 treatment effectively inhibited LD biogenesis in 1% O_2_ LCLs, confirming its on-target effects (**Extended Data** Fig. 4b). Notably, A922500 selectively impaired the growth of 1% O_2_ LCLs from multiple donors, while having minimal impact on 21% O_2_ LCLs (Fig. 5g **and Extended Data** Fig. 4c). Moreover, A922500 treatment selectively increased Caspase 3/7 activity in 1% O_2_ LCLs, further supporting the critical role of TG synthesis and LD formation in 1% O_2_ LCL survival (**Extended Data** Fig. 4d).

### Extracellular unsaturated fatty acids are essential for the survival of 1% O_2_ LCLs.

**Figure 6:**
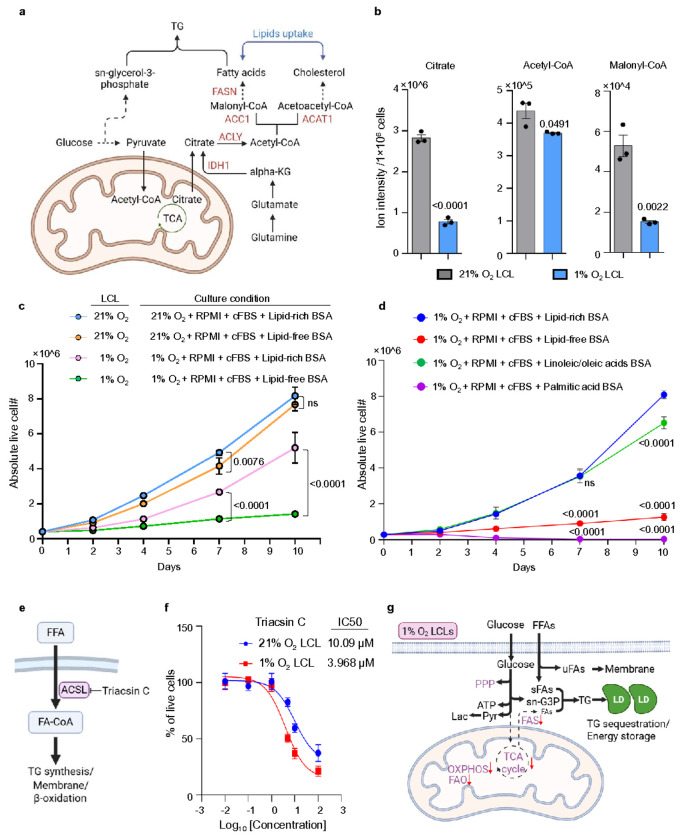
Extracellular unsaturated fatty acids are essential for the survival of 1% O_2_ LCLs. a. A schematic picture showing fatty acid synthesis and mevalonate pathway. b. Bar chart analysis of normalized RNAseq reads of indicated genes in 1% or 21% O_2_ LCLs. Mean +/− SD values were from n=3 experiments. P-values were calculated using an unpaired Student’s t-test. c. Growth curve of 1% O_2_ LCLs and 21% O_2_ LCLs cultured in indicated condition. Abbreviations in the culture condition: 21% O_2_, 21% O_2_ incubator; 1% O_2_, 1% O_2_ incubator; RPMI, RPMI-1640 media; cFBS, 10% charcoal-stripped FBS; Lipid-rich BSA, 1mg/mL; Lipid-free BSA, 1mg/mL. Mean +/− SD values are from n=3 experiments. P-values were calculated using two-way ANOVA with Tukey’s multiple comparisons test. d. Growth curve of 1% O_2_ LCLs cultured in indicated condition. LCLs derived from different donors are plotted separately in the extended fig. Abbreviations in the culture condition: 1% O_2_, 1% O_2_ incubator; RPMI, RPMI-1640 media; cFBS, 10% charcoal-stripped FBS; Lipid-rich BSA, 1mg/mL; Lipid-free BSA, 1mg/mL; Linoleic/oleic acids BSA, 1mg/mL; Palmitic acid BSA, 0.36 mg/mL. Mean +/− SD values are from n=3 experiments. P-values were calculated using two-way ANOVA with Dunnett’s multiple comparisons test. e. Schematic representation of fatty acid activation and its metabolic fates. Triacsin C, an ACSL inhibitor. f. IC50 analysis showing the effect of Triacsin C treatment on 1% O_2_ and 21% O_2_ LCLs. Mean +/− SD values are from n=3 experiments. g. Schematic of metabolic adaptations under hypoxia. FFA, free fatty acid; uFA, unsaturated fatty acid; sFA, saturated fatty acid, Lac, lactate; Pyr, pyruvate; sn-G3P, sn-glyceral-3-phosphate; LD, lipid droplet.

Previous research identified that mevalonate and fatty acid synthesis (FAS) pathways were amongst the most highly EBV induced^18^. Cholesterol and fatty acid biosynthesis begins with citrate, which is exported from mitochondria or synthesized in the cytosol via the reductive carboxylation of α-ketoglutarate (α-KG) catalyzed by isocitrate dehydrogenase 1 (IDH1). Citrate is converted to acetyl-CoA by ATP citrate lyase (ACLY). Acetyl-CoA is then carboxylated to malonyl-CoA by acetyl-CoA carboxylase (ACC1, encoded by *ACACA*), a rate-limiting enzyme in fatty acid synthesis. Malonyl-CoA and acetyl-CoA are subsequently utilized for fatty acid elongation, facilitated by fatty acid synthase (FASN) and related enzymes. For cholesterol biosynthesis, acetoacetyl-CoA, derived from acetyl-CoA, enters the mevalonate pathway, which produces key intermediates for cholesterol and other isoprenoid compounds essential for membrane structure and intracellular trafficking (Fig. 6a).

Our RNA-seq analysis revealed significant downregulation of *ACLY*, *ACACA*, *FASN*, and *IDH1* in 1% O_2_ LCLs compared to 21% O_2_ LCLs (**Extended Data** Fig. 5a). Consistently, metabolomic analysis showed a marked reduction in key FAS intermediates, including citrate, acetyl-CoA, and malonyl-CoA, while sn-glycerol-3-phosphate, a precursor for triglycerides and phospholipids, was significantly elevated in 1% O_2_ LCLs (Fig. 6b and 4g). Newly synthesized saturated fatty acids can be cytotoxic under hypoxic conditions, particularly when desaturation (via SCD1) and FAO are suppressed. Therefore, the observed downregulation of FAS genes and metabolites in 1% O_2_ LCLs likely reflects an adaptive response to limit lipotoxicity and maintain cellular homeostasis in the hypoxic environment.

The suppression of FAS in 1% O_2_ LCLs suggests that these cells must obtain lipids from alternative sources to sustain membrane synthesis and energy storage during the rapid proliferation. We hypothesized that 1% O_2_ LCLs rely on external fatty acids to meet their metabolic demands. To test this, we cultured 1% and 21% O_2_ LCLs in lipid-rich or lipid-low media. While 21% O_2_ LCLs proliferated regardless of external lipid availability, 1% O_2_ LCLs exhibited significantly impaired growth in lipid-low conditions. Supplementation with lipid-rich bovine serum albumin (BSA conjugated with linoleic, oleic, palmitic, stearic acids) partially rescued their growth, underscoring their dependence on external lipids for survival under hypoxia (Fig. 6c). Interestingly, linoleic/oleic acid BSA supplementation were sufficient to restore growth, whereas palmitate-BSA exacerbated cell death (Fig. 6d). This metabolic dependency was consistently observed across 1% O_2_ LCLs from multiple donors, suggesting a conserved adaptation to hypoxic stress (**Extended Data** Fig. 5b). Furthermore, palmitate-BSA supplementation rapidly increased caspase 3/7 activity, indicating acute induction of apoptosis (**Extended Data** Fig. 5c). In contrast, fatty acid-free media triggered caspase 3/7 activation more gradually, suggesting a slower but progressive onset of cell death due to lipid deprivation (**Extended Data** Fig. 5c).

When free fatty acids enter the cytosol, they are activated by acyl-CoA synthetase long-chain family members (ACSLs) to form fatty acyl-CoA, which serves as a substrate for various lipid metabolic pathways (Fig. 6e). To further examine the contribution of external fatty acid uptake, we treated 1% and 21% O_2_ LCLs with Triacsin C, a pan-ACSL inhibitor^76^. Notably, 1% O_2_ LCLs exhibited increased sensitivity to Triacsin C, with an IC_50_ of 3.968 μM, compared to 10.09 μM in 21% O_2_ LCLs, suggesting a heightened reliance on ACSL activity for lipid metabolism under hypoxia (Fig. 6f). These findings reinforce the notion that 1% O_2_ LCLs rely on external unsaturated fatty acids for survival.

### Mevalonate pathway remains critical for protein prenylation in 1% O_2_ LCLs.

RNAseq analysis revealed that acetyl-CoA acetyltransferase 1 (*ACAT1*), an enzyme critical for the condensation of acetyl-CoA into acetoacetyl-CoA in the early steps of the mevalonate pathway, was significantly downregulated in 1% O_2_ LCLs, indicating potential suppression of the pathway under hypoxia (Fig. 6a, **Extended Data** Fig. 6a). In contrast, the expression of low-density lipoprotein receptor (LDLR), a main cholesterol transporter, remained unchanged, suggesting that cholesterol uptake from extracellular sources may compensate for reduced cholesterol biosynthesis (**Extended Data** Fig. 6a). Consistent with this, metabolomics analysis showed that while total cholesterol levels were unaffected, there was a significant reduction in acetoacetyl-CoA, a precursor in the mevalonate pathway, in 1% O_2_ LCLs. This supports the idea that external cholesterol uptake via LDLR maintains cellular cholesterol levels under hypoxic conditions (**Extended Data** Fig. 6b).

Protein prenylation is essential for EBV-transformed LCLs, with geranylgeranylation playing a key role in small GTPase activation, including Rab and Rho family members, which regulate intracellular trafficking, cytoskeletal dynamics, and signal transduction^18^. Geranylgeranyl pyrophosphate (GGPP), a crucial product of the EBV-induced mevalonate pathway, activates Rab13, facilitating LMP1 and LMP2A trafficking and signaling that are necessary for GM12878 LCL survival and proliferation^18^. To assess the role of this pathway in 1% O_2_ LCLs, we treated cells with simvastatin, an HMG-CoA reductase (HMGCR) inhibitor, which blocked the mevalonate pathway (**Extended Data** Fig. 6c). Both 1% and 21% O_2_ LCLs were highly sensitive to simvastatin, exhibiting significant growth defect, highlighting the conserved role of the mevalonate pathway in LCL survival (**Extended Data** Fig. 6d). Notably, supplementation with GGPP rescued the growth of 1% O_2_ LCLs, consistent with previous findings in GM12878 LCLs^18^ (**Extended Data** Fig. 6e-g). These results strongly suggest that the primary function of the mevalonate pathway in LCLs is to supply intermediates for protein prenylation, reinforcing the critical dependency of LCLs on geranylgeranylation to sustain EBV oncogenic signaling, regardless of oxygen levels.

## Discussion

The hypoxic transformation of EBV-infected B-cells reveals a unique set of metabolic adaptations that diverge significantly from conventional transformation models (Fig. 6g). These adaptations underscore the role of hypoxia as a selective pressure that reshapes transformation physiology to sustain proliferation and survival under oxygen-limited microenvironments.

Beyond the well-known metabolic shift toward glycolysis, a critical distinction between EBV transformation at 21% O_2_ and 1% O_2_ involves lipid metabolism. While 21% O_2_ LCLs activate FAS driven by EBNA2, MYC, and SREBPs^18^, 1% O_2_ LCLs notably express lower levels of FAS. The mechanism underlying FAS suppression in 1% O_2_ LCLs remains unclear. One potential explanation comes from recent work by Li et al., who showed that limited NAD^+^ availability—due to impaired electron acceptor capacity under hypoxia or electron transport chain inhibition—can constrain FAS^77^. In our study, we observed an increase in total NAD^+^ levels in 1% O_2_ LCLs, yet the NAD^+^/NADH ratio remained unchanged compared to 21% O_2_ LCLs. It is plausible that hypoxia may lead to a shift in compartmental NAD^+^ pools in EBV transformed B-cell. A reduced mitochondrial NAD^+^ pool could directly limit the lipogenic citrate, thereby contributing to FAS downregulation. It is also important to note that, unlike studies that utilize established cancer cell lines, our model captures an active oncogenic transformation process that occurs under hypoxic conditions. This prolonged transformative process under hypoxia likely engages additional layers of regulation—including transcriptional and epigenetic mechanisms—that modulate metabolism beyond the immediate effects of redox imbalance.

Given the suppression of FAS, 1% O_2_ LCLs must rely on alternative lipid sources to sustain proliferation and membrane biogenesis. Our data indicates that these cells compensate by increasing the reliance on external unsaturated fatty acids, such as oleic and linoleic acids, which are well-tolerated and support cell growth. In contrast, exposure to saturated fatty acids like palmitic acid is highly toxic in LCLs. To further mitigate lipotoxic stress, 1% O_2_ LCLs exhibit elevated TG storage and prominent LD formation — features that suggest a protective mechanism for sequestering excess saturated lipids. This buffering system appears to be regulated in part by HIF-1α-induced genes, notably HILPDA, which is upregulated 13.2-fold under hypoxia. HILPDA has been shown to promote lipid droplet formation by enhancing DGAT activity and inhibiting adipose triglyceride lipase (ATGL), thereby stabilizing TG accumulation and mitigating lipotoxicity^78–80^.

Interestingly, when 1% O_2_ LCLs are re-exposed to 21% O_2_, they demonstrate an even greater proliferative capacity than 21% O_2_ LCLs. We speculate that they retain intact mitochondrial function and are capable of reactivating FAO using lipids stored as TGs for energy production and biosynthesis. This metabolic flexibility may provide a survival advantage, potentially contributing to the aggressiveness of GC-derived lymphomas that transition from hypoxic to oxygen-rich microenvironments.

We observed that hypoxia induces profound alterations in redox metabolism. Under hypoxic conditions, the upregulation of the PPP supports NADPH production, enhancing the ability of transformed cells to mitigate excessive ROS. However, EBV infection triggers substantial ROS production under conventional B-cell transformation models, which plays a pivotal role in causing DNA damage necessary for cell transformation and subsequent immortalization into LCLs^81^. The critical involvement of ROS in EBV-mediated B-cell immortalization is underscored by the observation that transformation is significantly impaired in the presence of ROS scavengers^81^. We speculate that physiologically relevant hypoxia may further modulate ROS dynamics during EBV-mediated transformation. This raises the possibility that hypoxia could predispose transformed cells to genomic instability, particularly in long-term residence within hypoxic microenvironments such as GCs. Such instability could facilitate the acquisition of oncogenic mutations, bridging the gap between EBV-mediated transformation and tumorigenesis, ultimately driving the progression from benign hyperproliferation to malignant growth. This hypothesis aligns with observations in EBV-associated malignancies, many of which originate in germinal center B-cells within the hypoxic microenvironment of secondary lymphoid tissues^24^. Hypoxia-induced genomic instability, coupled with the pro-survival and growth-promoting effects of EBV oncoproteins such as LMP1, could create a fertile ground for the emergence of oncogenic mutations. Future investigations are needed, particularly given the well-documented interplay between elevated ROS levels and impaired DNA repair mechanisms under hypoxic conditions in cancer contexts^82^.

Despite hypoxia’s established effects on chromatin accessibility, we did not observe significant alterations in EBV-driven SEs at key loci such as MYC and IRF4. This stability suggests that hypoxia-adaptive pathways are layered onto a robust chromatin framework established by viral oncogenes. However, the mechanisms through which the EBV oncogenic program modulates hypoxia adaptation remain to be fully elucidated.

EBV intrinsically drives metabolic reprogramming by leveraging HIF-1α signaling, with its effects evident even under normoxic conditions, where LMP-driven HIF-1α activation and EBNA3A/LP-mediated HIF-1α stabilization contribute to glycolytic gene expression^83–85^. Under hypoxia, however, HIF-1α activation is markedly amplified, synergizing with the EBV oncogenic program to confer metabolic plasticity to LCLs. This heightened HIF-1α activity under low oxygen not only enhances glycolytic metabolism but also drives the expression of angiogenic and extracellular matrix remodeling genes, such as *VEGFA* and *P4HA1*, equipping cells with the ability to thrive in and adapt to hypoxic microenvironments^86^. The interplay between hypoxia-induced HIF-1α activation and EBV-driven pathways, including NF-κB and PI3K/AKT, fortifies pro-survival and growth-promoting mechanisms, underscoring the critical role of hypoxia in shaping the metabolic and oncogenic landscape of EBV-transformed cells.

In conclusion, hypoxia orchestrates a distinct transformation program in EBV-infected B-cells, marked by profound metabolic reprogramming to prioritize glycolysis, lipid remodeling, and redox balance, promoting survival, proliferation, and potentially tumorigenesis. These findings provide a framework for exploring the intersection of viral oncogenesis, hypoxic adaptation, and tumor progression, offering new insights into the vulnerabilities of EBV-associated malignancies. For example, inhibitors of DGAT or lipid uptake pathways may selectively impair hypoxic-transformed cells in the GCs.

## Materials and Methods

### Human Primary B cells isolation

De-identified human whole blood samples were obtained from Research Blood Components, LLC (Watertown, MA, USA) with approval from IRB: 120160613. All samples were prescreened and confirmed negative for common human pathogens. As the samples were de-identified, donor gender was unknown. Studies on primary human blood cells were approved by the Tufts University Institutional Review Board (Tufts IRB: STUDY00004385). Primary human B-cells were isolated by negative selection using RosetteSep and EasySep Human B-Cell Enrichment kits (Stem Cell Technologies) following the manufacturers’ protocols. B-cell purity was confirmed by CD19 plasma membrane expression via flow cytometry. Cells were cultured in RPMI 1640 medium (Gibco) with 10% fetal bovine serum (FBS, F31016-500, SeraPrime).

### EBV production and concentration

The EBV B95-8 strain was generated from B95-8 cells engineered for inducible ZTA expression (a generous gift from Dr. Ben Gewurz). The activation of EBV lytic cycle was achieved by treating the cells with 1 μM of 4-hydroxytamoxifen (4HT, Sigma-Aldrich) for 24 hours. Subsequently, the 4HT was removed, and the cells were cultured in RPMI medium supplemented with 10% FBS, devoid of 4HT, for an additional 96 hours. The viral supernatants obtained were then cleared of producer cells by passing through a 0.45 μm filter. The supernatant was transferred to an ultracentrifuge tube (326823, Beckman Coulter) and centrifuged at 25,000 rpm for 2 h at 4°C in an ultracentrifuge (OPTIMA XPN-100, Beckman Coulter). The viral pellet was resuspended and aliquoted in PBS with 2% dialyzed FBS, stored at −80°C until infection. The genomic DNA of virus was quantified by PCR targeting the BALF5 gene from the extracted viral genome as described^87^. This quantification was used to standardize the virus amounts for cell infection experiments.

### EBV hypoxic transformation model

Purified naïve B-cells were incubated with B95-8 EBV at a multiply of infection (MOI) of 0.1 for 1 hour at room temperature to facilitate viral entry. Following infection, cells were washed with serum free RPMI-1640 medium and resuspended in complete culture medium (RPMI-1640 supplemented with 10% FBS, 2 mM L-glutamine, 100 U/mL penicillin, and 100 μg/mL streptomycin). To assess the impact of oxygen levels on EBV transformation, infected B-cells were cultured under two distinct oxygen conditions: 1) normoxia (21% O_2_): cells were maintained in a standard tissue culture incubator (Thermo Fisher Scientific) at 37°C with 5% CO_2_. The oxygen level in the incubator was confirmed to be 21%, consistent with atmospheric oxygen at sea level (Boston). 2) hypoxia (1% O_2_): cells were placed in a hypoxic incubator (Thermo Fisher Scientific) set to 1% O_2_, 5% CO_2_, and 94% N_2_ at 37°C. Cells were cultured for 28 days, during which they proliferated and transformed into LCLs. LCLs were passaged every 3 days by replacing 2/3 of the medium with fresh RPMI-1640 supplemented with 10% FBS to support cell growth.

### Cell viability and growth analysis

#### 1. Cell viability and growth analysis

Cell viability was assessed using the Countess 3 Automatic Cell Counter (Thermo Fisher Scientific) with Trypan Blue staining (15250061, Thermo Fisher Scientific) to distinguish live from dead cells. For growth curve analysis, live cell counts were recorded at each time point.

To evaluate oxygen-dependent growth dynamics, LCLs from each donor were split into two flasks and cultured in RPMI-1640 medium supplemented with 10% FBS under either 1% or 21% O_2_ in dedicated hypoxic or normoxic incubators, respectively.

#### 2. Lipid supplementation studies

To assess lipid dependency, charcoal-stripped fetal bovine serum (cFBS; A3382101, Gibco) was used to reduce external fatty acid levels. While not completely delipidated, cFBS contains significantly lower levels of free fatty acids and lipophilic molecules compared to regular FBS, allowing controlled lipid supplementation experiments. LCLs cultured under 1% or 21% O_2_ were washed twice with dPBS and maintained in RPMI-1640 medium containing 10% cFBS supplemented with either 1 mg/mL lipid-rich BSA (11020039, AlbuMAX^™^ I, Gibco) or 1 mg/mL lipid-free BSA (A8806-1G, Sigma-Aldrich).

To define specific lipid requirements under hypoxia, 1% O_2_ LCLs were cultured in RPMI-1640 + 10% cFBS supplemented with: (i) 1 mg/mL lipid-rich BSA, (ii) 1 mg/mL lipid-free BSA, (iii) 1 mg/mL oleic/linoleic acid–conjugated BSA (L9655, Sigma-Aldrich), or (iv) 0.36 mg/mL palmitic acid–conjugated BSA (29558, Cayman).

#### 3. Mevalonate pathway inhibition

To probe the role of the mevalonate pathway, LCLs under both oxygen conditions were treated with 2 μM simvastatin (S1792, Selleckchem) or DMSO control. For rescue experiments, 1% O_2_ LCLs were additionally supplemented with 2 μM geranylgeranyl pyrophosphate (GGPP; G6025, Sigma-Aldrich) to assess the requirement for protein prenylation. At the start point, cells were seeded at 3×10^5^/mL. Additives were refreshed upon cell splitting.

#### 4. Inhibiting TG Biosynthesis

To assess the role of triglyceride biosynthesis and lipid droplet formation in the survival of LCLs under 1% O_2_, cells cultured under both normoxic and hypoxic conditions were treated with 10 μM A922500 (HY-10038, MedChemExpress) or DMSO as a control. At the start point, cells were seeded at 3×10^5^/mL. Treatments were refreshed with 10 μM A922500 at 24 and 48 hours post the initial treatment. At 72 hours, cells were counted and harvested for Caspase-3/7 activity analysis.

#### 5. Inhibiting super-enhancers using THZ1

21% and 1% O_2_ LCLs were treated with 100 nM THZ1 (HY-80013, MedChemExpress) or DMSO for 4 days under respective oxygen conditions. At the start point, cells were seeded at 3×10^5^/mL. Cell viability was assessed using trypan blue staining followed by automatic cell counting using a Countess 3 cell counter.

#### 6. 6-Aminonicotinamide treatment

1% or 21% O_2_ LCLs were treated with DMSO or 100 μM 6-Aminonicotinamide, a G6PD inhibitor, (6-AN, No.S9783, Selleckchem) for 48 hours. At the start point, cells were seeded at 3×10^5^/mL. Cell viability was assessed using trypan blue staining followed by automatic cell counting using a Countess 3 cell counter.

#### 7. Triacsin C treatment.

To determine the IC_50_ of Triacsin C (BML-EI218, Enzo) in 21% and 1% O_2_ LCLs, cells were seeded into 96-well plates and treated with a range of Triacsin C concentrations (0.001 μM to 100 μM) alongside DMSO-only controls. Plates were incubated at 37°C under their respective oxygen conditions for 72 hours, after which cell viability was assessed using trypan blue staining followed by automatic cell counting using a Countess 3 cell counter. Viability was normalized to the DMSO control, and data were plotted as percentage of live cells versus the log_10_ of Triacsin C concentration. IC_50_ values were calculated by fitting a sigmoidal dose-response curve using nonlinear regression.

For all the growth curve analysis, to avoid overconfluency and ensure accurate growth analysis, cultures were regularly split, and total live cell numbers were adjusted based on dilution factors at each passage. Unless otherwise noted, LCLs were consistently maintained in incubators matching their designated oxygen conditions (1% or 21% O_2_).

### Chromatin Immunoprecipitation (ChIP)

Histone H3K27ac and H3K4me3 ChIP-seq in 1% O_2_ and 21% O_2_ LCLs was performed using the iDeal ChIP-seq kit for Histones (C01010059, HILOGIC Diagenode) following the manufacturer’s protocol, with ChIP-grade antibodies listed in Supplementary Table S1. DNA libraries were prepared using the NEBNext^®^ Ultra^™^ II DNA Library Prep Kit for Illumina (E7645S, NEB) and sequenced at the Tufts Genomics Core. Read quality was assessed using FastQC to ensure no biases such as GC skew or PCR artifacts. ChIP-seq reads were aligned to the human genome (hg19) using default settings using Bowtie2, except -k was set to 1, with a mappability rate of 94–98%^88^. Peaks were called using MACS v2.1.074 with an FDR threshold of ≤ 0.99^89^, followed by IDR analysis (v2.0.3) with an IDR threshold of ≤ 0.02, as recommended by the ENCODE consortium to ensure peak reproducibility^90^. Peaks in blacklist regions were excluded from downstream analysis. Super-enhancers (SEs) were identified using HOMER under default settings, and ChIP-seq heatmaps were generated using deepTools v3.5.6^91^.

### RNAseq analysis

Total RNA was isolated by the RNeasy Mini kit (Qiagen), following the manufacturer’s manual. An in-column DNA digestion step was included to remove the residual genomic DNA contamination. To construct indexed libraries, 1 μg of total RNA was used for polyA mRNA-selection, using the NEBNext Poly(A) mRNA Magnetic Isolation Module (New England Biolabs), followed by library construction via the NEBNext Ultra RNA Library Prep Kit (New England Biolabs). Each experimental treatment was performed in triplicate. Libraries were multi-indexed, pooled and sequenced on an Illumina NextSeq 500 sequencer using single-end 75 bp reads (Illunima) at the Dana Farber Molecular Biology core. Adaptor-trimmed Illumina reads for each individual library were mapped back to the human GRCh37.83 transcriptome assembly using STAR2.5.2b ^92^. Feature Counts was used to estimate the number of reads mapped to each contig ^93^. Only transcripts with at least 5 cumulative mapping counts were used in this analysis. DESeq2 was used to evaluate differential expression (DE) ^94^. DESeq2 uses a negative binomial distribution to account for overdispersion in transcriptome datasets. It uses a conservative analysis that relies on a heuristic approach. Each DE analysis used pairwise comparison between the experimental and control groups. Differentially expressed genes were identified and a *P*-values < 0.05 and absolute fold change > 2 cutoff was used. Differentially expressed genes (DEGs) were subjected to Enrichr analysis which was employed to perform gene list-based gene set enrichment analysis on the selected gene subset. The algorithm used to calculate combined scores was described previously ^95^. P value and log_2_ fold change were generated with DESeq2 under default settings with Wald test and normal shrinkage, respectively. Top 5 Enrichr terms that passed the adjusted p-value cutoff were visualized using Graphpad Prism 7. Volcano plots were built with Graphpad Prism7. To concentrate on differentially regulated metabolic pathways, DEGs enriched in 1% O_2_ LCLs were filtered with a curated metabolic gene list^56^ and subjected to the STRING protein-protein interaction networks and functional enrichment analysis^96^.

### Lipidomic profiling analysis

Lipid profiling was performed as described previously^97, 98^. Briefly, 1% and 21% O_2_ LCLs were counted and pelleted at 1,200 rpm for 5 minutes at 4 °C with an equal number of cells in each sample. They were then resuspended in 200 μL of HPLC-grade water (270733, Sigma-Aldrich) and mixed vigorously with 2.5 mL of HPLC-grade methanol (A456, Fisher Scientific) in glass tubes. Following this, 5 mL of methyl tert-butyl ether (MTBE, 1634-04-4, Supelco) was added, and the samples were agitated for 1 hour at room temperature. To separate phases, 1.5 mL of water was added, and after vigorous vortexing, the samples were centrifuged at 1000 x g for 10 minutes at room temperature. The upper phase was then dried a speed vacuum concentrator (Savant SPD 1010, Thermo Fisher Scientific) for 4 h at RT and stored at −80 °C.

For analysis, samples were reconstituted in 35 μL of a 1:1 mixture of LCMS-grade isopropanol and methanol, and subjected to liquid chromatography-mass spectrometry (LC-MS) as previously outlined, employing a high-resolution hybrid QExactive HF Orbitrap mass spectrometer (Thermo Fisher Scientific) set to data-dependent acquisition mode (Top 8) with the capability of switching between positive and negative ion polarities. Lipid species identification and quantification were performed using the LipidSearch 4.1.30 software (Thermo Fisher Scientific), leveraging an internal database comprising ≥20 major lipid classes and ≥80 subclasses. For verifying signal linearity, a pooled sample was created by combining 5 μL from each sample, which was then diluted with a 1:1 mixture of isopropanol and methanol to generate dilutions of 0.3x and 0.1x, alongside a blank. These dilutions underwent analysis, and for each lipid species within this series, the Pearson correlation coefficient between ion count and sample concentration was computed. Only lipids exhibiting a correlation coefficient (r) greater than 0.9 were retained for final analysis. The abundance of individual lipid species was normalized against the total ion count of the sample. Using R, lipids were categorized by class, and the total ion intensity for each lipid class in each sample was calculated.

### Intracellular metabolite profiling

The intracellular metabolites profiling was performed as described^99^. 1% and 21% O_2_ LCLs were washed 3 times with pre-chilled PBS and counted. The cell pellet was fully resuspended with 100uL PBS by vortex, the metabolism was quenched by adding 3.3 mL of dry ice-cold 80% aqueous methanol (A456, Fisher Scientific), and kept at −80°C overnight. The lysate was centrifuged at 21,000 g for 15 min at 4°C. The supernatants were obtained and dried by a speed vacuum concentrator (Savant SPD 1010, Thermo Fisher Scientific) for 4 hours at RT. Samples were re-suspended using 20 uL HPLC grade water for mass spectrometry. 5-7 μL were injected and analyzed using a hybrid 6500 QTRAP triple quadrupole mass spectrometer (AB/SCIEX) coupled to a Prominence UFLC HPLC system (Shimadzu) via selected reaction monitoring (SRM) of a total of 300 endogenous water soluble metabolites for steady-state analyses of samples. Some metabolites were targeted in both positive and negative ion mode for a total of 311 SRM transitions using positive/negative ion polarity switching. ESI voltage was +4950V in positive ion mode and −4500V in negative ion mode. The dwell time was 3 ms per SRM transition and the total cycle time was 1.55 seconds. Approximately 9-12 data points were acquired per detected metabolite. Samples were delivered to the mass spectrometer via hydrophilic interaction chromatography (HILIC) using a 4.6 mm i.d x 10 cm Amide XBridge column (Waters) at 400 μL/min. Gradients were run starting from 85% buffer B (HPLC grade acetonitrile) to 42% B from 0-5 minutes; 42% B to 0% B from 5-16 minutes; 0% B was held from 16-24 minutes; 0% B to 85% B from 24-25 minutes; 85% B was held for 7 minutes to re-equilibrate the column. Buffer A was comprised of 20 mM ammonium hydroxide/20 mM ammonium acetate (pH=9.0) in 95:5 water:acetonitrile. Peak areas from the total ion current for each metabolite SRM transition were integrated using MultiQuant v3.0.2 software (AB/SCIEX). Metabolites with p-values < 0.05, log2(fold change)>1 or <−1 were used for pathway analysis using MetaboAnalyst 5.0 (https://www.metaboanalyst.ca/MetaboAnalyst/ModuleView.xhtml).

### Seahorse mitochondrial stress test

The Agilent Seahorse XF Assay was conducted as described preiously^14^. Specifically, the sensor cartridge was first hydrated with water overnight and incubated with XF Calibrant for 1h. Add 12 μL Cell-Tak solution (1.3 mL of 0.1M sodium bicarbonate, 11.2 μL of 0.1M NaOH, 22.4 μL of Cell-Tak solution) to each well of the V7-PS 96-well cell culture plate. The Cell-Tak solution was washed with sterile water twice and 0.25 million 1% and 21% O_2_ LCLs (resuspension in 180 μL of RPMI-1640 with 10% FBS and 5 mM pyruvate) were seed on a Seahorse plate. Then the cells were placed in a non-CO_2_ 37 °C for 30 minutes. The oxygen consumption rates (OCR) and extracellular acidification rate (ECAR) were simultaneously recorded by a Seahorse XFe96 Analyzer (Agilent). The cells were sequentially probed by 20 μL of 3.5 μM oligomycin A (No.S1478, Selleckchem), 20 μL of 2 μM CCCP (No.S6494, Selleckchem), and 20 μL of 100 nM piericidin A (HY-114936, MedChemExpress). Data was analyzed by Seahorse Wave Desktop Software (Agilent).

### Flow cytometry analysis

The mitochondrial mass was determined by the MitoTracker Green FM (M7514, Thermo Fisher Scientific) following the manual. 1×10^6^ of Cells were collected and resuspended in 500 μL cell culture media with 1.5 μL 100 μM of MitoTracker Green. Cells were then incubated in 37°C incubator for 30 min. Then cells were washed once with 1×PBS and resuspended in PBS buffer with 2% FBS for FACS. For CFSE (C345544, Invitrogen) cell proliferation staining, 10 million of primary B-cells were resuspended in PBS with 0.1% BSA, then the cells were mixed with the same volume of 1μM CFSE for 10 min at 37°C. Cells were then neutralized by prechilled 10% FBS RMPI-1640 for 5 min. After washing the cell with culture media, cells were resuspended and infected with EBV. 1 h after infection, cells were treated with 1% or 21% O_2_ for 5 or 7 days. As a control, CFSE stained primary B-cells were stimulated with a combination of 1μg/mL anti-human IgM IgG (I0759, Sigma-Aldrich) and 0.5 μM CpG (T*C*G*T*C*G*T*T*T*T*G*T*C*G*T*T*T*T*G*T*C*G*T*T, IDT) and cultured in a 1% or 21% O_2_ incubator for 5 days. Flow cytometry was performed on a BD FACS Calibur instrument. Data was analyzed with FlowJo V10.

### Caspase activation assay

Caspase 3/7 activity was quantified by Caspase-Glo assays (G8090, Promega) according to manufacturer’s manual and normalized to the cell number of the same sample determined by Trypan Blue staining and cell counting using an automatic cell counter Countess 3 (Thermo Fisher Scientific). All values were quantitated on a Promega^™^ GloMax^®^ Plate Reader (Promega).

### Western blot analysis

Immunoblot analysis was performed according to the previous methods^100^. Cell lysates were prepared by incubating cells in 1× Laemmli buffer at 95 °C for 5 min. Lysate Samples were separated by SDS-PAGE electrophoresis, transferred onto the nitrocellulose membranes, blocked with 5% milk in TBST buffer for 1 h, and then probed with relevant primary antibodies at 4°C overnight. Restore^™^ Western Blot Stripping Buffer (21063, Thermo Fisher Scientific) was used when necessary. The next day, the membranes were incubated with secondary antibody for 1 h. Blots were then developed by incubation with ECL chemiluminescence (Millipore) and images were captured by Licor Fx system. Bands intensities were measured where indicated by Image Studio Lite Version 5.2. All antibodies used in this study were listed in Supplementary Table S1.

### Confocal microscopy

1% O_2_ LCLs, 21% O_2_ LCLs treated with DMSO or 10 μM DGAT1 inhibitor, A922500 (HY-10038, MedChemExpress) for 24 hours were supplemented with 10 μM Bodipy FL C12 (D3822, Thermo Fisher Scientific) for 30min. Cells were then washed once with PBS and resuspended in RMPI-1640 with 10% charcoal-stripped FBS for live cell confocal imaging with Zeiss LSM900. To ensure precise timing after Bodipy FL C12 treatment, experiments were conducted one cell line at a time.

### Quantification and statistical analysis

Unless otherwise indicated, all bar graphs and line graphs represent the arithmetic mean of three independent experiments (n = 3), with error bars denoting standard deviations. Data were analyzed using unpaired Student t-test or analysis of variance (ANOVA) with the appropriate post-test using GraphPad Prism7 software. Gene ontology analysis was done with the Enrichr module using the KEGG pathway databases. Default parameters of Enrichr module was used, with the exception that the Enrichment statistic was set as classic. Metabolic pathway analysis was performed using MetaboAnalyst 6.0. Figures were drawn with commercially available GraphPad, Biorender, Microsoft Powerpoint.

### Data availability

All RNA-seq and ChIP-seq datasets have been deposited to the NIH GEO omnibus. The accession number for the RNA-seq dataset reported in this paper is GSE293238. The deposited dataset will be released upon acceptance. All plasmids and cell lines generated in this study will be made available on request. Other ChIP-seq or ChIA-PET data were obtained from GEO: GM12878 LCL POLR2A ChIA-PET, GSE72816; H3K4me3 ChIP-seq, GSE95899; H3K27ac ChIP-seq, GSM733771.

### Graphics

Figures were drawn with GraphPad, Biorender, Microsoft Powerpoint, and ggplot2 in R.
